# From adiposity to steatosis: metabolic dysfunction-associated steatotic liver disease, a hepatic expression of metabolic syndrome – current insights and future directions

**DOI:** 10.1186/s40842-024-00187-4

**Published:** 2024-12-02

**Authors:** Bruno Basil, Blessing K. Myke-Mbata, Onyinye E. Eze, Augustine U. Akubue

**Affiliations:** 1 Department of Chemical Pathology, Benue State University, Makurdi, Nigeria; 2 Department of Nursing, Central Washington College, Enugu, Nigeria; 3 Department of Chemical Pathology, Bingham University, Jos, Nigeria; 4 Department of Haematology and Blood Transfusion, Enugu State University of Science and Technology, Enugu, Nigeria

**Keywords:** Adiposity, Metabolic syndrome, Metabolic dysfunction-associated steatotic liver disease (MASLD), Non-alcoholic fatty liver disease (NAFLD), Obesity, Steatosis

## Abstract

**Background:**

Metabolic dysfunction-associated steatotic liver disease (MASLD) is a growing health concern and the risk of its development is connected with the increasing prevalence of metabolic syndrome (MetS) which occurs as a result of some complex obesity-induced metabolic changes. It is a common chronic liver disease characterized by excessive fat accumulation in the liver, the tendency to progress to more severe forms, and a corresponding increase in morbidity and mortality. Thus, effectively addressing the rising burden of the disease requires a thorough understanding of its complex interrelationship with obesity and MetS.

**Main Body:**

MASLD results from complex interactions involving obesity, insulin resistance, and dyslipidaemia, leading to hepatic lipid accumulation, and is influenced by several genetic and environmental factors such as diet and gut microbiota dysbiosis. It has extensive metabolic and non-metabolic implications, including links to MetS components like hyperglycaemia, hypertension, and dyslipidaemia, and progresses to significant liver damage and other extra-hepatic risks like cardiovascular disease and certain cancers. Diagnosis often relies on imaging and histology, with non-invasive methods preferred over liver biopsies. Emerging biomarkers and OMIC technologies offer improved diagnostic capabilities but face practical challenges. Advancements in artificial intelligence (AI), lifestyle interventions, and pharmacological treatments show promise, with future efforts focusing on precision medicine and novel diagnostic tools to improve patient outcome.

**Conclusion:**

Understanding the pathogenic mechanisms underlying the development of MASLD within the context of metabolic syndrome (MetS) is essential for identifying potential therapeutic targets. Advancements in non-invasive diagnostic tools and novel pharmacological treatments, hold promise for improving the management of MASLD. Future research should focus on precision medicine and innovative therapies to effectively address the disease and its consequences.

## Background

Accumulation of excessive adipose tissue in obesity initiates a chain of complex metabolic changes which contributes to the development of metabolic syndrome (MetS). This interplay of metabolic disturbances fuels the progression of obesity and MetS, setting the stage for the emergence of non-alcoholic fatty liver disease (NAFLD), which constitutes a substantial public health challenge [1]. The excess visceral fat in obesity and the systemic consequences of MetS synergistically lead to insulin resistance which promotes the accumulation of triglycerides in the liver [2]. In turn, NAFLD which encompasses a spectrum from benign steatosis to more severe forms with inflammation and fibrosis emerges as the hepatic manifestation of this metabolic imbalance. The interplay of insulin resistance, inflammatory mediators, and dysregulated lipid metabolism forms the common pathophysiologic relationship between obesity, MetS, and NAFLD [3], and a good understanding of these concepts would lead to more effective management outcome.

Obesity is a chronic, progressive, relapsing and treatable multi-factorial neurobehavioral condition, characterized by an accumulation of excess body fat and resulting in adverse metabolic, biomechanical, and psychosocial health consequences [4]. It is estimated that approximately 1.9 billion adults worldwide are overweight, from which over 650 million adults were obese. Overall, approximately 13% of the world’s adult population (11% of men and 15% of women) was obese in 2016, and these figures are increasing. More worrying is the increasing trend of obesity in children and adolescents [5]. A major consequence of obesity is the development of MetS and its accompanying interrelated comorbidities including NAFLD, type 2 diabetes mellitus (T2DM), cardiovascular disease (CVD), hypertension, obstructive sleep apnoea (OSA), hyperlipidaemia, chronic kidney disease (CKD), osteoarthritis (OA) and malignancies (e.g., breast, colon and prostate) amongst others, leading to increased mortality [6].

NAFLD is characterised by excessive fat accumulation in the liver, greater than 5% hepatocytes, in the absence of other competing liver aetiologies including excess alcohol intake, chronic viral hepatitis and the use of medications that induce steatosis [7]. Recently, due to a better understanding of its mechanisms, the name has been changed to metabolic dysfunction-associated steatotic liver disease (MASLD) although not without certain implications [8, 9]. It is the commonest cause of chronic liver disease with a pooled global prevalence of 38.2% (95% CI: 33.7 – 42.9) as at 2019 with the highest regional prevalence in Latin America (44.4%) and North-Africa and Middle-East (36.5%) [10], progressing to non-alcoholic steatohepatitis (NASH), in about 20% of cases [11]. The prevalence of NAFLD is estimated to be 75.3% (95% CI: 70·9–79·2) in the obese population, 70.0% (95% CI: 65·4–74·2) in overweight people, and 10.6% (95% CI: 7.8–14.1) amongst normal weight individuals [12, 13]. Due to the increasing incidence of obesity in children and adolescents, NAFLD is now increasingly diagnosed amongst children at prevalence rates of 4.6% to 9.0% with a yearly increase of 0.26% and forecast analysis predicting a prevalence of 30.7% by 2040 [14].

NAFLD is a general term describing the histological spectrum ranging from non-alcoholic fatty liver (NAFL) which is marked by lipid buildup in hepatocytes to non-alcoholic steatohepatitis (NASH) involving inflammation. The severity of NASH can range significantly between individuals, with varying levels of fibrosis, cirrhosis and hepatocellular carcinoma (HCC) which may culminate in hepatic decompensation and death [15]. The rising global prevalence of NAFLD corresponding to the increasing burden of obesity has spurred extensive research into the complex pathogeneses of the disease in relation to obesity and MetS, as well as its clinical implications and management strategies. Exploring NAFLD as the liver’s expression of MetS helps in the identification of shared metabolic pathways which may enhance risk assessment and guide clinical management with significant public health implications.

This review aims to update and enhance the knowledge of MASLD, with emphasis on its association with MetS and obesity for clinicians and researchers, facilitating more effective strategies for the diagnosis, risk assessment, and management of individuals affected by these interconnected metabolic conditions. It was conducted through a detailed search of scientific literature using online databases including PubMed, Google Scholar, and Web of Science. Keywords such as "NAFLD," "Metabolic Syndrome," "MASLD," "obesity," "pathogenesis," "clinical implications," "diagnosis," "interventions," and related terms were used to identify relevant recent articles published up to the present date. Studies including original research articles, reviews, meta-analyses, and clinical trials addressing the interplay between NAFLD, MetS, and obesity were included. Additionally, recent advancements in diagnostic modalities, therapeutic interventions, and emerging biomarkers were explored to provide insights into future directions for NAFLD management.

## Pathogenesis of NAFLD

### Overview of the underlying molecular mechanisms

Fat accumulation in the liver results from an impairment in the balance between lipid acquisition and lipid disposal. These processes are tightly regulated by four major pathways involving complex interactions between hormones, nuclear receptors, and transcription factors. They include uptake of circulating lipids and de novo lipogenesis (DNL) – lipid acquisition; and fatty acid oxidation (FAO) and export of lipids in very low-density lipoproteins (VLDL) – lipid disposal (Table 1) [16–21].

**Table 1:** Molecular Mediators involved in the Pathogenesis of NAFLD

Pathogenic Pathways	Molecular Mediator	Role in the Pathogenesis of NAFLD
Uptake of Circulating Lipids	Fatty Acid Transport Proteins (FATP)	Mediates the hepatic uptake of circulating free fatty acids (FFAs), contributing to about 60% of triglyceride (TG) influx into the liver (26% – DNL, unhealthy diets – 15%). Genetic alterations in FATP5 promotor suggest a potential link to BMI-dependent hepatic steatosis
	Cluster of Differentiation 36 (CD36)	Facilitates the transport of long-chain fatty acids and is regulated by peroxisome proliferator-activated receptor (PPAR) γ, pregnane X receptor, and liver X receptor. Increased CD36 expression in NASH, along with translocation from the cytoplasm to the plasma membrane during NAFLD progression, implicates its role in hepatic lipid accumulation
	Caveolins	Although the role of caveolin 1 in NAFLD is not fully understood, studies suggest a potential decrease in its expression following a high-fat diet, indicating a complex relationship with lipid metabolism
	Fatty Acid-Binding Proteins (FABP)	Elevated levels of hepatic FABPs (FABP1, FABP4, and FABP5) in NAFLD patients suggest increased intracellular trafficking of fatty acids, potentially promoting steatosis by enhancing their transport within the lipid-laden liver
De Novo Lipogenesis (DNL)	Sterol Regulatory Element-Binding Protein 1c (SREBP1c)	Orchestrates the transcriptional regulation of DNL, activated by insulin and liver X receptor α. Dysregulation in NAFLD, particularly during transitions from fasting to the fed state, suggests a central role in hepatic lipid accumulation
	Carbohydrate Regulatory Element-Binding Protein (ChREBP)	ChREBP is activated by carbohydrates, and contributes to upregulation of glycolytic enzymes and fatty acid synthase thereby promoting hepatic DNL. Adiposity and insulin resistance in NAFLD patients result in liver overload of glucose and insulin, activating ChREBP and SREBP-1c
	IL-1β Signaling	May contribute fat accumulation in the liver by debilitating the insulin sensing pathways, disrupting normal lipid metabolism and activating inflammatory pathways
Fatty Acid Oxidation (FAO)	Peroxisome Proliferator-Activated Receptor Alpha (PPARα)	Controls FAO and is crucial in preventing hepatic lipid accumulation. Downregulation is associated with NAFLD progression, impacting both lipid homeostasis and inflammation
	Oxidative Stress and Inflammation	Oxidative stress, generated during lipid overload and exacerbated by enzymes CYP2E1 and CYP4A11, diminishes mitochondrial function and potentially upregulates peroxisomes as a compensatory response
Export of Lipids in VLDL	Apolipoprotein B100 (apoB100)	Plays a pivotal role in VLDL formation and export and is positively regulated by PPARα. Genetic defects compromise triglyceride export
	Microsomal Triglyceride Transfer Protein (MTTP)	Key player in VLDL formation and export, also positively regulated by PPARα but negatively regulated by insulin. Genetic defects compromise triglyceride export contributing to hepatic steatosis. Increased VLDL secretion in NAFLD patients, while initially compensatory, may plateau with high intrahepatic lipid content, possibly contributing to lipid retention and disease progression

*apoB100* Apolipoprotein B100, *BMI* Body Mass Index, *CD*36 Cluster of Differentiation 36, *ChREBP* Carbohydrate Regulatory Element-Binding Protein, *DNL* De Novo Lipogenesis, *FABP*, Fatty Acid-Binding Proteins, *FAO* Fatty Acid Oxidation, *FATP* Fatty Acid Transport Proteins, *FFAs* Free Fatty Acids, *IL-1β* Interleukin-1 Beta, *MTTP* Microsomal Triglyceride Transfer Protein, *NASH* Non-Alcoholic Steatohepatitis, *PPARα* Peroxisome Proliferator-Activated Receptor Alpha, *ROS* Reactive Oxygen Species, *SREBP1c* Sterol Regulatory Element-Binding Protein 1c, *VLDL* Very Low-Density Lipoproteins

### Role of visceral adiposity and insulin resistance

Visceral adiposity, the hallmark of metabolic syndrome, plays a key role in the development of Insulin Resistance (IR) which in turn leads to development of NAFLD. Following accumulation of excessive body fat, particularly in the viscera, adipose tissue dysfunction and altered adipose metabolic processes play a fundamental role in the development of IR both in lean and obese individuals [1]. This is characterised by low adiponectin levels, high TNF-alpha and other pro-inflammatory cytokines like interleukin-6 secretion from the adipose organ, as well as enhanced lipolysis with serum elevations of free fatty acids (FFAs). These factors jointly initiate changes in numerous modulators of insulin sensing pathways, including but not limited to alterations in insulin receptor substrate (IRS) proteins, protein kinase B (Akt), and c-Jun N-terminal kinase (JNK), resulting in the development of IR [22, 23].

IR plays a major role in the development of NAFLD by promoting hepatic lipogenesis (due to hyperinsulinaemia, as this pathway retains its sensitivity to insulin) and impairing the inhibition of adipose tissue lipolysis, subsequently leading to the accumulation of fats in the liver mainly as triglycerides, resulting in NAFL. This is considered a defensive response rather than a hepatotoxic event, aiming to counterbalance excessive FFAs in the bloodstream [24]; However, the actions of other bioactive intermediates like ceramides and diacylglycerol (DAG) induce lipotoxicity, contributing to inflammation, necrosis, and fibrosis [25]. Ceramides, for example, activate stress kinases such as JNK and inhibit Akt, promoting hepatic lipid accumulation and insulin resistance, while DAG activates protein kinase C (PKC), leading to impaired insulin signaling and increased gluconeogenesis [26, 27].

Furthermore, adipose tissue macrophages, which are recruited to adipose tissue in obesity, also play a critical role in promoting inflammation and insulin resistance through the secretion of pro-inflammatory cytokines and adipokines such as TNF-alpha and interleukin-1 beta [28]. These inflammatory mediators act in a paracrine and endocrine manner, promoting hepatic inflammation and fibrogenesis, further exacerbating NAFLD progression [29].

### Role of dyslipidaemia and hypertension

The primary sources of fatty acids influx into the liver are the systemic plasma FFAs, originating from the lipolysis of the adipose tissue TG, and DNL in the liver from simpler precursors, e.g., carbohydrates [26]. This DNL from simpler precursors then lead to further increase in systemic FFAs which contributes to both adipose tissue expansion and hepatocytes fat deposition (NAFLD) driving both processes simultaneously. Rarely, some individuals exhibit a genetically predisposed lipodystrophic phenotype characterised by increased lipolysis which overwhelms the body's ability to store lipids subcutaneously leading to FFA accumulation in visceral areas of the body, including in the liver. This errant lipid metabolism drives IR and inflammation and is similar but unrelated to NAFLD progression in the more classic obesity-driven and T2DM-driven pathogenesis [30].

Although the exact mechanisms underlying the association between hypertension (HTN) and NAFLD is still unclear, recent studies present new evidence suggesting that elevated blood pressure levels, even within the normal range, could predict the onset of NAFLD [31]. The putative mechanism involves HTN triggering activation of the renin–angiotensin–aldosterone system (RAAS) and the sympathetic nervous system (SNS). This activation, in turn, is believed to stimulate insulin resistance, induce hepatic inflammation, and ultimately contribute to the development NAFLD. A bidirectional relationship between NAFLD and HTN have been established, and it appears to be independent of classical cardiometabolic risk factors. The closed two-way link between NAFLD and HTN can form a vicious circle during disease progression [31], and further studies suggest that blood pressure control may help reduce the risk of developing NAFLD [32].

### Role of genetic and environmental factors

The development of NAFLD is influenced by a combination of factors, including environmental cues like dietary habits and physical activity, as well as inherited factors such as genetic and epigenetic influences. Additionally, the intestinal microbiota and its by-products are recognized as crucial contributors to the pathophysiology of NAFLD [33].

Some genetic alterations have been implicated in the pathogenesis of NAFLD. Genetic variants, particularly single nucleotide polymorphisms (SNP), influence various processes related to NAFLD development and progression, such as FFAs flow into the liver, oxidative stress, response to endotoxins, and cytokine production [34]. The SNP of the patatin-like phospholipase domain-containing protein 3 (PNPLA3) gene's I148M variant is implicated in NAFLD [35], showing associations with reduced de novo lipogenesis, increased expression of SREBP-1c, and higher risks of steatosis and liver fibrosis [36]. Another variant, rs58542926, in the transmembrane 6 superfamily member 2 (TM6SF2) gene, is linked to lower plasma VLDL levels, hepatic steatosis, and higher ALT levels [35, 37]. These genes along with other protective variants in hydroxysteroid 17-beta dehydrogenase 13 (HSD17B13), mitochondrial amidoxime reducing component 1 (MARC1) and cell death-inducing DFFA-like effector B (CIDEB) can be integrated to form polygenic risk scores which are associated with outcomes such as liver fat accumulation, cirrhosis, and the risk of hepatocellular carcinoma [38].

Some other genetic variants that have been implicated in NAFLD include variants in the MBOAT7 gene which encodes an enzyme involved in the remodelling of phospholipids, glucokinase regulatory protein (GCKR) gene which regulates glucose metabolism by modulating the activity of glucokinase, apolipoprotein C3 (APOC3) gene involved in the regulation of triglyceride metabolism, lipoprotein lipase (LPL) gene coding for an enzyme involved in the hydrolysis of triglycerides in circulating lipoproteins, and adiponectin receptor 2 (ADIPOR2) gene which modulates adiponectin signaling and insulin sensitivity. These genetic variants contribute to the pathogenesis of NAFLD through various mechanisms, including alterations in lipid metabolism, glucose homeostasis, and adipokine signaling [35, 39–42].

Certain epigenetic modifications represent stable transcriptional changes, like DNA methylation, histone modifications, and microRNA (miRNA) activity. These are involved in maintaining cellular equilibrium by exhibiting a significant level of adaptability influenced by environmental changes, and a disturbance in this balance has been suggested to increase ones vulnerability to NAFLD [43].

Also, dietary shortage of methyl group donors like betaine, choline, and folate, affects DNA methylation a key determinant which leads to NAFLD, and betaine supplementation is associated with reduced methylation of the MTTP promoter, with the subsequent increase in triglycerides efflux from the liver [44]. Also, folate deficiency impacts FFA synthesis gene expression, and reduced expression/activity of SIRT1, a deacetylase protein, is linked to NAFLD [45]. Additionally, non-coding microRNAs (miRNAs) regulate epigenetic gene expression, and changes in their expression are associated with NAFLD and MASH pathogenesis. For instance, MiR-122, highly expressed in the liver, influences plasma cholesterol levels and liver gene expression related to cholesterol and fatty acid synthesis [46].

The role of diet in NAFLD is influenced by both caloric content and nutrient types. Overeating is linked to hepatic steatosis and steatohepatitis risk, with certain substrates being more steatogenic. For instance, fructose, metabolized into fructose-1-phosphate, acts as a pro-inflammatory lipogenic factor, contributing to oxidative stress and TNF-α overproduction [47]. Fructose-induced NAFLD is associated with bacterial proliferation, increased intestinal permeability, and liver fibrosis, potentially mediated by hepatic ATP depletion [48]. High-calorie drinks with sucrose elevate the risk of liver steatosis and NASH [49]. Conversely, coffee and monounsaturated fats along with moderate alcohol (red wine) intake (typical Mediterranean diet) have been shown to have hepato-protective effects, possibly due to antioxidants and caffeine [50].

The gut microbiota emerges as a critical player in NAFLD, influencing both its development and progression. Studies reveal dysbiosis in NAFLD patients, showcasing alterations in microbial composition with disease progression. Notably, obese juvenile NAFLD patients exhibit increased concentrations of *Gammaproteobacteria* and *Prevotella*, with increase in *Proteobacteria* and decrease in *Firmicutes *observed as NAFLD advances [51]. Dysbiosis, characterized by translocation, may enhance gut permeability, facilitating increased fatty acid absorption. This heightened permeability leads to bacterial migration and the release of toxic products like lipopolysaccharides (LPS) and proinflammatory cytokines, initiating and perpetuating inflammation. The Toll-like receptor 4 (TLR-4)-mediated activation of Nuclear-Factor-kappa-B (NF-κB) plays a pivotal role in this process [52]. The gut–liver axis further accentuates the impact on hepatic tissue, given its sensitivity to the influx of blood through the portal vein [53]. Additionally, the intestinal microbiota's influence on bile acid metabolism, modulating farnesoid X receptor (FXR) stimulation, contributes to NAFLD pathogenesis by affecting de novo lipogenesis and VLDL export processes [54].

## Clinical implications of NAFLD

### Metabolic manifestations

Recent studies have shown that increased number of MetS components in NAFLD patients correlate with an increased risk of overall, and liver-related mortality [55], and increase in the risk of T2DM associated with NAFLD [56]. Conversely, strong evidence indicate that NAFLD is independently associated with increased risk of development of MetS and T2DM which parallels its severity, emphasizing the interconnected nature of these conditions [1, 57].

In NAFLD, excess hepatic fat accumulation leads to hepatic insulin resistance, resulting in increased hepatic glucose production which exacerbates hyperglycaemia. This state of chronic hyperglycaemia contributes to systemic insulin resistance, further promoting hepatic lipid accumulation and worsening NAFLD [58]. Inflammatory mediators released from the liver and adipose tissue, such as TNF-alpha and IL-6, further exacerbate insulin resistance and create a pro-inflammatory environment that fuels both NAFLD and T2DM [59]. These pathogenic mechanisms in the bi-directional relationship between NAFLD and T2DM presents a viscous cycle which exacerbates the course of each disease [60]. NAFLD amplifies insulin requirements, microvascular complications, cardiovascular risk, and mortality in diabetic patients [61], making it a major consideration in their management. A similar bidirectional relationship occurs between incidence and progression of NAFLD and hypertension [31] which increases all cause and cardiovascular mortality [62].

Another typical manifestation in these patients is atherogenic dyslipidaemia, notably elevated fasting serum triglycerides, apo-B, small dense LDL (sdLDL), and reduced HDL-cholesterol, independently of insulin resistance [63]. Dyslipidaemia is linked to the severity of NAFLD, worsening from NAFL to NASH, but paradoxically improves on progression to advanced fibrosis and cirrhosis as steatosis disappears [64], possibly due to impaired hepatic synthetic capacity. In NASH, elevated serum non-HDL cholesterol levels persist even after NASH resolution while triglycerides and HDL improves [65].

Additionally, NAFLD presents as a significant risk factor for incident hyperuricemia and has a bidirectional relationship with it [66]. Hyperuricemia, often overlooked in conventional metabolic syndrome criteria, demonstrates a strong association with the severity of steatosis and progression to fibrosis in NAFLD patients [67].

### Hepatic manifestations

The progression of NAFLD has significant consequences on liver health. The risk of liver-related mortality increases exponentially with each stage of fibrosis, as demonstrated in a meta-analysis of biopsy-proven NAFLD patients [15]. The progression of NAFLD from simple steatosis (or NAFL) involves a pathophysiological cascade which begins with the intra-hepatic fat accumulation [17]. This pathological hallmark of NAFL is often marked by the presence of PNPLA3 I148M polymorphism, a genetic variant associated with NAFLD progression which influences lipid metabolism, contributing to further increase in hepatic fat accumulation [20, 68].

As NAFLD advances, the transition to NASH is marked by inflammation, hepatocyte injury, and immune cell recruitment. Inflammatory pathways, notably NF-κB, play a pivotal role in perpetuating hepatocellular damage alongside IL-6 and TNF-α [25, 69]. The subsequent development of fibrosis is a critical feature in the progression of NAFLD. Hepatic stellate cells (HSCs) are activated by pro-inflammatory cytokines and profibrogenic signals, with transforming growth factor-beta (TGF-β) signaling playing a central role. This activation leads to the deposition of extracellular matrix, resulting in fibrosis [69]. The extent of fibrosis serves as a determinant of disease prognosis, and advanced fibrosis can culminate in cirrhosis. Progression of NASH to cirrhosis is generally slow, though varying among individual patients and probably more dynamic than previously thought [70]. NASH patients may experience spontaneous fluctuations, demonstrating both progression and regression of their liver disease over a long period of time [71].

Furthermore, chronic inflammation, oxidative stress, and sustained activation of cellular pathways create an environment conducive to carcinogenesis. Molecular alterations in one-carbon metabolism, NF-κB activation, and dysregulated microRNA expression contribute to the hepatocarcinogenic process [72]. The progression to HCC is influenced by factors such as the severity of fibrosis and the presence of cirrhosis, with specific genetic variants like PNPLA3 and HSD17B13 contributing to the increased risk [73].

### Extra-hepatic conditions associated with NAFLD

Non-metabolic extra-hepatic conditions associated with NAFLD encompass cardiovascular risks, including atherogenic dyslipidaemia and hypertension, contributing to coronary artery disease, endocrine disorders, etc. NAFLD is also linked to chronic kidney disease, certain cancers, particularly colorectal cancer, and neurological issues such as cognitive impairment and neuropathy. These highlights the systemic impact and diverse health risks associated with this liver disorder as shown in various studies (Table 2) [74–79].

**Table 2:** **Extra-hepatic conditions associated with NAFLD** [74–79]

Organ Systems	Associated Disease Entity	Comments
Cardio-vascular	Atherosclerosis	NAFLD is strongly associated with carotid artery atherosclerosis, and incident CV events
	Structural Heart Diseases (AVS, MAC, Cardio-myopathy and HF)	The significant association between NAFLD and Cardiovascular complications is particularly pronounced in patients with more severe histological forms of NAFLD, indicating a strong rationale for the development of cardiomyopathy and heart failure amongst them
	Arrhythmias	NAFLD is associated with an increased risk of AF and VA. It can also can predict AF independent of T2DM and other conventional cardiometabolic comorbidities
	CAD	NAFLD is significantly correlated with cardiovascular outcomes of CAD, irrespective of traditional CVD risk factors
	CKD	There is a robust association between NAFLD and CKD, particularly in individuals with more severe forms of NAFLD
Endocrine	Hypercortisolism and hypogonadism	It is possible that a bidirectional association between hypercortisolism and hypogonadism, and the NAFLD pathway exists. Further studies required
Respiratory	OSAS and COPD	Establishing an association between NAFLD and the development of OSAS and COPD is challenging due to the presence of various comorbidities which contribute to systemic inflammation. Additional studies are required
Musculo-skeletal	Osteoporosis and osteopenia	A causal association between NAFLD and development of osteoporosis and osteopenia both in adults and in children may exists but it is uncertain. Further studies required
	Sarcopenia	Presence of a possible bidirectional relationship between NAFLD/NASH and development of sarcopenia is uncertain. Further studies required
	Periodontitis	A potential association between NAFLD and periodontitis may be due to the effect of other co-existing metabolic disorders. There is also a moderate bidirectional causal impact of NAFLD on periodontitis
Skin	Psoriasis	Available evidence supports a bidirectional association between NAFLD and psoriasis, especially in patients below the age of 40 years. Further studies are required to prove causation
Gastro-intestinal	GERD	Often associated with MetS, and there is increased risk of GERD even in the absence of obesity. A possible bidirectional relationship may occur
Tumours	Colorectal adenoma and carcinoma	Patients with NAFLD may have an increased risk of colorectal tumours compared with those without NAFLD
	Pancreas, Gastric, Prostate, Breast, Oesophageal, and others	Increased risk of these cancer types has been attributed to NAFLD. It is also associated with moderately increased long-term risk of developing extra-hepatic cancers (especially GI cancers, breast cancer and gynaecological cancers)
Psycho-logical dysfunction	MDD and others e.g., cognitive impairment, Alzheimer’s, bipolar disorder, schizophrenia etc	The exact pathophysiologic connection between NAFLD and MDD is not well-established. There is a significant reduction in both white and grey matter volumes in the brains of individuals with NAFLD compared to control subjects indicating and increased risk for developing MDD, as well as in other types of psychological dysfunction, such as cognitive impairment and Alzheimer’s disease, and bipolar disorder and schizophrenia. A bidirectional relationship is also suggested

*AF* Atrial fibrillation, *AVS* Aortic-valve sclerosis, *CAD* Coronary Artery Disease, *CKD* Chronic kidney disease, *COPD* Chronic obstructive pulmonary disease, *CV* Cardiovascular, *GERD* Gastrointestinal reflux disease, *GH* Growth Hormone, *HF* Heart failure, *HTN* Arterial hypertension, *MAC* Mitral annular calcification, *MDD* Major depressive disorder, *MetS* Metabolic syndrome, *NAFLD* Non-alcoholic fatty liver disease, *OSAS* Obstructive sleep apnea syndrome, *PCOS* Polycystic ovary syndrome, *T2DM* Type 2 diabetes mellitus, *VA* Ventricular Arrythmia

## Diagnosis and Assessment of NAFLD

### Current approaches

Screening for NAFLD is recommended for patients with metabolic risk factors. It involves demonstrating fatty liver disease through imaging or histology, ensuring the absence of other chronic liver diseases, excessive alcohol consumption, and secondary steatosis. Preferred screening methods include liver ultrasound to confirm steatosis, along with fibrosis scores, liver function tests, and elastography techniques to identify potential candidates for liver biopsy, particularly those at risk of advanced disease [80].

Diagnosis of NAFLD is often by abdominal ultrasound, a popular and useful technique [81]. However, liver biopsy is considered the gold standard providing valuable information regarding disease activity, grading, and staging. The clinical utility of liver biopsy is limited by the length and location of the biopsy as it captures only approximately 1/50,000, of the total liver tissue, hence the controversy surrounding its status as the gold standard for diagnosis [82]. Also, the high and increasing prevalence of NAFLD and the risks associated makes liver biopsy unsuitable for initial and routine assessment. This necessitates the use of non-invasive diagnostic tools for comprehensive assessment of NAFLD and its progression [83]. NASH takes approximately seven years to advance by one fibrosis stage, while those with simple steatosis require 14 years for the same progression [20]. In cases where patients’ fibrosis test results are unremarkable but risk factors persist, it is recommended that the tests is repeated every three years to monitor and assess liver health [84]. Various scoring systems and imaging modalities are available for this purpose (Table 3) [84–86] instead of relying solely on imaging methods which are particularly confounded by a range of conditions including infiltrative liver disease, liver congestion, acute hepatitis, liver inflammation and cholestasis [86].

**Table 3:** Some Scoring Systems and Imaging Modalities for Evaluation and Monitoring of NAFLD Progression [84–86]

Test	Parameters	Interpretation	Evaluation
*Quantification of steatosis*			
Fatty Liver Index (FLI)	BMI, waist circumference, GGT, triglycerides	FLI < 30 = exclusion of steatosis (sensitivity 87%) FLI > 60 = steatosis diagnosed (specificity 86%)	Inexpensive, easy to determine; Moderate accuracy; suitable for large epidemiological studies
Hepatic Steatosis Index (HSI)	ALT, BMI, Sex, and Diabetes status	A higher index suggests a higher probability of hepatic steatosis	Inexpensive, easy to determine; also suitable for large epidemiological studies
"Controlled Attenuation Parameter" (CAP)	Integrated into the FibroScan® device	Cut-off values: Steatosis Grade 1 when CAP ≥ 302 dB/m (AUROC 87%) Steatosis Grade 2 when CAP ≥ 331 dB/m (AUROC 77%) Steatosis Grade 3 when CAP ≥ 337 dB/m (AUROC 70%)	Exact determination of the degree of steatosis not possible Interpretation limited in higher-grade obesity Suitable for assessing progression in known NAFLD
*Assessment of high-risk NASH*			
Fibrotic NASH Index (FNI)	AST, HDL, HbA1c	Rule-out (Sensitivity): 0.1 (89%), Rule-in (Specificity): 0.33 (90%), AUROC = 0.78	Assesses for fibrotic NASH NPV was 93%; PPV was 57% It is also a marker for presence and severity of NASH
NIS-4	α2-macroglobulin, YKL-40, HbA1c, miR34a	Rule-out (Sensitivity): < 0.36 (82%), Rule-in (Specificity): > 0.63 (87%), AUROC = 0.80	It is also a marker for presence and severity of NASH
MRI cT1	MRI-based calculation	Rule-out (Sensitivity): < 825 ms (78%) Rule-in (Specificity): ≥ 875 ms (90%) AUROC = 0.78	It is also a marker for presence and severity of NASH
*Diagnosis of NASH*			
NASH Test	13 parameters (age, sex, height, weight, triglycerides, total cholesterol, alpha-2-macroglobulin, apolipoprotein A1, haptoglobin, GGT, ALT, AST, total bilirubin)	Three categories: (1) NASH – 79% diagnostic accuracy (2) Borderline NASH – 69% diagnostic accuracy (3) No NASH – 77%–83% diagnostic accuracy	Mainly used in specialized liver centers; no general recommendation
Cytokeratin-18 (CK-18) fragments	M30 and M65	CK-18 fragments released from apoptotic hepatocytes and measured directly in blood Detection of CK-18 reflects inflammatory liver injury in NASH Several studies identified a cut-off for M30 of ≥ 200 U/L for the diagnosis of NASH	Lack of a uniform cut-off value for NASH detection In the risk assessment of NAFLD patients, CK-18 or other CK-18 based scores can be used
*Fibrosis assessment*			
Fibrosis-4 Index (FIB-4)	Age, platelet count, AST and ALT	FIB-4 < 1.3 = advanced fibrosis excluded (sensitivity 74%) FIB-4 > 3.25 = advanced fibrosis diagnosed (specificity 98%)	Easy to determine, inexpensive Recommended to exclude advanced liver fibrosis (F3/F4) in NAFLD Serial FIB-4 predicts liver-related outcomes
AST-to-platelet ratio index (APRI)	AST and Platelet count	< 0.5 – greater NPV (rule out cirrhosis) > 1.5 – greater PPV (rule in cirrhosis) Midrange values are less helpful	Easy to interpret; Relatively inexpensive; Limited by modest accuracy
AST-to-ALT ratio	AST, ALT	AUROC 0.66–0.74 for F3 fibrosis (Sensitivity—40%; Specificity—80%)	Easy to interpret; Limited by modest accuracy and reproducibility
NAFLD Fibrosis Score (NFS)	Age, BMI, abnormal fasting glucose/diabetes mellitus, AST/ALT quotient, platelet count, albumin	NFS < –1.455 advanced fibrosis excluded (sensitivity 90%) NFS > 0.676 advanced fibrosis diagnosed (specificity 97%)	Recommended to exclude advanced liver fibrosis (F3/F4) in NAFLD Less accurate < 40 years; less specific > 60 years
BARD score	AST, ALT, BMI and Diabetes mellitus	AUROC 0.69–0.81 for F3 fibrosis (Sensitivity 62%, Specificity 66%)	High, as common parameters involved Interpretation of BMI might differ across different ethnic groups
FibroSpect	Hyaluronic Acid (HA), Tissue Inhibitor of Metalloproteinase-1 (TIMP-1), α2-Macroglobulin	Utilizes a proprietary algorithm that combines HA, TIMP-1, and α2-Macroglobulin as parameters Specific numerical values or ranges for interpretation (e.g., < 16) are not reported in the provided information	Proprietary test – details of the algorithm may not be publicly disclosed Easy to interpret results
FibroTest	GGT, total bilirubin, α2-microglobulin, apolipoprotein AI and haptoglobin	0.31 – 0.48 (Stage F1-F2) – Minimal fibrosis 0.72 – 0.74 (Stage F3-F4) – Advanced fibrosis Non- binary AUROC for fibrosis 0.88	Suboptimal for early- stage fibrosis Useful in different chronic liver disease; accurate in patients with overweight or obesity
FibroMeter NAFLD	Body weight, prothrombin index, ALT, AST, ferritin and fasting glucose	AUROC 0.76 for F2 fibrosis (Sensitivity—22%, Specificity—97%); 0.77 for F3 fibrosis (Sensitivity—27%, Specificity—95%)	High cost Accurate for severe fibrosis in different liver diseases
FIB-C3	Age, BMI, T2DM (binary variable), Platelet count and PRO-C3	< − 0.4 suggests higher risk; > − 0.4 suggests lower risk	Requires non-routine clinical data; lower NPV Uses a single threshold for risk stratification
ELF (Enhanced Liver Fibrosis)	Hyaluronic Acid, N-terminal propeptide of type III procollagen, Tissue Inhibitor of Metalloproteinases	< 7.7 indicates risk; > 9.8 suggests lower risk	Easy interpretation; High NPV May be costly
Vibration Controlled Transient Elastography (VCTE) FibroScan®	From the velocity of the reflected shear waves (in m/s), the liver stiffness (in Kpa) can be calculated Mechanically induced impulse	Threshold value depend on the aetiology of chronic liver disease Cut-off values: 8.2 kPa for F ≥ 2, Sensitivity 85%, specificity 70% 9.7 kPa for F ≥ 3, Sensitivity 71%, specificity 75% 13.6 kPa for F4, Sensitivity 85%, specificity 79%	Suitable for exclusion of advanced hepatic fibrosis and cirrhosis Point of care; high NPV Multiple potential confounders; requires experienced operator In obese patients (BMI > 30 kg/m2), limitations noted with standard M probe
2D-shear wave elastography	Ultrasound induced radiation force focus swept over depth faster than shear wave speed to create a Mach cone Quantitative measurement of shear wave speed	AUROC 0.85–0.92 for F2 fibrosis (Sensitivity 85%, specificity 94%, PPV 94%, NPV 85%) AUROC 0.88–0.95 for F3 fibrosis (Sensitivity 90%, specificity 92%, PPV 88%, NPV 93%) AUROC 0.97 for F4 fibrosis (Sensitivity 100%, specificity 86%, PPV 55%, NPV 100%)	Implemented on a regular USS machine; Enables simultaneous Sonographic imaging of the liver Requires fasting for 2-h; Experienced operators needed; Quality criteria not well defined
Point-shear wave elastography	Ultrasound induced focused radiation force impulse at death Quantitative measurement of shear wave speed	• AUROC 0.70–0.83 for F2 fibrosis (Sensitivity 56–90%, specificity 36–90%) • AUROC 0.74–0.97 for F3 fibrosis (Sensitivity 59–90%, specificity 63–90%) • AUROC 0.78–0.89 for F4 fibrosis (Sensitivity 44–90%, specificity 67–90%)	Implemented on a regular ultrasonography machine. Enables simultaneous sonographic imaging of the liver Requires fasting for 2-h. Quality criteria not well defined
MR Elastography (MRE)	Uses a modified phase- contrast method to image the propagation of the shear wave in the liver parenchyma	> 3.64 kPa – high diagnostic accuracy for advanced fibrosis (AUROC 92%); few confounders	Expensive, requires MRI facility (limited availability); Time consuming and costly High concordance with histological severity

*ALT* Alanine Aminotransferase, *apoB100* Apolipoprotein B100, *AST* Aspartate Aminotransferase, *AUROC* Area Under the Receiver Operating Characteristic Curve, *BMI* Body Mass Index, *cT1* Contrast-Enhanced T1, *CAP* Controlled Attenuation Parameter, *CK-18* Cytokeratin-18 Fragments, *DNL* De Novo Lipogenesis, *ELF* Enhanced Liver Fibrosis, *FABP* Fatty Acid-Binding Protei, *FAO* Fatty Acid Oxidation, *FATP* Fatty Acid Transport Proteins, *FIB-4* Fibrosis-4 Index, *FibroSpect* FibroSpect, *FibroTest* FibroTest, *FNI* Fibrotic NASH Index, *GGT* Gamma-Glutamyl Transferase, *HA* Hyaluronic Acid, *HDL* High-Density Lipoprotein, *HSI* Hepatic Steatosis Index, *IL-1β* Interleukin-1 Beta, *M30* Cytokeratin-18 Fragment M30, *M65* Cytokeratin-18 Fragment M65, *miR34a* microRNA 34a, *MRI* Magnetic Resonance Imaging, *MRE* MR Elastography, *MTTP* Microsomal Triglyceride Transfer Protein, *NASH* Nonalcoholic Steatohepatitis, *NFS* NAFLD Fibrosis Score, *NIS-4* NAFLD Inflammatory Score-4, *NPV* Negative Predictive Value, *PPAR* Peroxisome Proliferator-Activated Receptor, *PPV* Positive Predictive Value, *ROS* Reactive Oxygen Species, *SREBP1c* Sterol Regulatory Element-Binding Protein 1c, *TG* Triglycerides, *TIMP-1* Tissue Inhibitor of Metalloproteinase-1, *USS* Ultrasonography, *VCTE* Vibration Controlled Transient Elastography (FibroScan®), *VLDL* Very Low-Density Lipoproteins

### Emerging biomarkers, OMIC technologies, and artificial intelligence

Emerging biomarkers are revolutionizing the assessment and understanding of Non-Alcoholic Fatty Liver Disease (NAFLD) and its progression. One such biomarker, FGF-21, shows promising potential in predicting NAFLD in individuals with Type 2 Diabetes Mellitus (T2DM) due to its high sensitivity and specificity compared to other markers [87]. Moreover, FGF-21 is garnering attention as a potential therapeutic target for obesity-related metabolic disorders, including NAFLD, owing to its significant effects on lipid and carbohydrate metabolism [88].

Elevated levels of circulating nucleosomes, particularly specific histones like macro H2A1.2, H2B, and H4, are associated with disease severity in NAFLD. Notably, the transition from NAFLD to NASH involves inflammation, with histone release associated with Neutrophil Extracellular Traps (NETs) contributing to this process [89]. Advanced DNA sequencing coupled with bioinformatics now enables the prediction of tissue-of-origin for circulating nucleosome-associated DNA, offering insights into differentially regulated pathways linked to liver conditions. However, the integration of such approaches into routine clinical practice faces challenges due to technical requirements and the need for trained personnel. Furthermore, the applicability of these approaches to high-risk groups, such as individuals with T2DM or metabolic syndrome, remains to be elucidated.

Several other new biomarkers have emerged in recent years to assess the activity and progression of NASH and liver fibrosis in patients with NAFLD [86, 90]. These biomarkers, including amino-terminal propeptide of type III procollagen (PIIINP), Pro collagen III (Pro-C3) hyaluronic acid and laminin, offer insights into collagen turnover, tissue repair, and active fibrogenesis. Tissue inhibitors of metalloproteinases (TIMPs) like the ELF panel provide valuable diagnostic and prognostic information, demonstrating excellent performance in discriminating patients with NASH-related fibrosis and predicting liver-related events and mortality. Novel molecular diagnostic tests like the combination of HOMA, AST, and CK18 (MACK-3) have been extensively evaluated and offer promising avenues for identifying high-risk NASH patients, while simpler scores such as Fibrotic NASH Index (FNI) show good performance for ruling out fibrotic NASH [91, 92]. Triglyceride glucose index–related parameters have been suggested as a potential effective early screening indicator for NAFLD and when combined with HOMA-IR, were more effective for evaluating metabolic risks and tracking disease progression in NAFLD patients [93]. Furthermore, Mac-2 binding protein glycan isomer (M2BPGi) has shown superiority in predicting fibrosis progression compared to some established scores [94].

However, implementation of these new biomarkers is limited by various challenges including high costs, limited accessibility in resource-poor areas, and the need for specialized equipment and trained personnel. Additionally, further validation and standardization are necessary to ensure reliability across diverse populations, and various confounding factors like comorbid conditions, medication use, and lifestyle variables can affect biomarker levels and their interpretation. Despite these problems, the development of NAFLD biomarkers holds significant promise for enhancing disease diagnosis, monitoring, and management, ultimately improving patient outcomes. Further validation and research are necessary to fully integrate these new markers into clinical practice.

Advancements in OMICs technologies have revolutionized the understanding and diagnosis of NAFLD. These high-throughput biological research methods like genomics, epigenomics, transcriptomics, metabolomics, proteomics, etc. are enhancing opportunities for better management of NAFLD. For instance, genomics studies have elucidated the role of common single nucleotide polymorphisms (SNPs) in NAFLD progression, paving the way for potential therapeutic interventions. Epigenomics investigations have highlighted the diagnostic potential of DNA methylation patterns, with specific markers showing promise in discriminating severe fibrosis and predicting the risk of T2DM. Additionally, micro-RNAs have emerged as key players in transcriptional regulation, with miR-34a-5p proving to be a discriminatory biomarker for at-risk NAFLD [95]. Recent advances in NAFLD metabolomics have further enriched diagnostic capabilities, offering panels of proteins and metabolic profiles that differentiate between NAFLD stages [90, 96].

Despite these advancements, the clinical translation of OMICs findings remains a challenge, necessitating further research and validation for widespread use in clinical practice. These include the high costs and need for sophisticated infrastructure which are prohibitive in resource-limited areas, the requirement for specialized bioinformatics expertise and standardized protocols to deal with the complexity and volume of OMICs data, and the need for extensive validation and standardization to ensure reliability across diverse populations amongst other challenges. Overcoming these barriers will require strategic investments in infrastructure, targeted training for healthcare professionals, collaborative research efforts, the development of clear regulatory frameworks, and the exploration of cost-effective approaches and technological innovations.

Presently, Artificial Intelligence (AI) plays a crucial role in pathology and imaging by providing objective assessment and minimizing inter- and intra-observer variability. In clinical practice, AI-assisted ultrasound and MRI are anticipated to improve diagnostic accuracy, particularly in the evaluation of diseases like NAFLD, offering the potential for objective assessment of histological features [81].

## Advancements in interventions for NAFLD and MetS

A comprehensive analysis involving various interventions for NAFLD and MetS patients revealed significant advancements and highlights the multi-dimensional approach required for effectively managing NAFLD in the context of metabolic syndrome. Studies evaluating dietary modifications, physical activity interventions, and metabolic and bariatric surgery demonstrate promising outcomes. Energy-restricted Mediterranean Diet (MD) combined with behavioural support effectively reduces BMI, waist circumference (WC), and glycaemic parameters [97]. Similarly, physical activity interventions exhibit benefits in reducing NAFLD incidence and waist circumference [98]. In other to enhance the effectiveness of these traditional methods of treatment of NAFLD, digital therapeutics (DTx) like mobile health applications and wearable devices are currently being introduced as a new method for the convenient management and treatment of patients with NAFLD, and are attracting a great deal of attention. DTx, which provide evidence-based medicine through software programs for remote intervention in preventing, treating, or managing diseases may overcome the drawbacks of traditional treatment. They provide medication reminders, guide rehabilitation, assess treatment outcomes, predict disease risks, and personalize management and treatment, which can greatly improve clinical work efficiency [99].

Metabolic and Bariatric surgery (MBS) has shown effectiveness in achieving long-term, sustained weight loss and has been widely documented to have beneficial effects in reversing obesity-related conditions that contribute to the development of NAFLD in obese individuals including T2DM. This has shown remarkable benefits in the management of NAFLD [100]. Notably, Roux-en-Y gastric bypass, results in substantial resolution of NASH and hepatic steatosis in severe obesity patients [101].

Currently, there very few FDA approved therapy for this disease and appropriate therapeutic targets are urgently warranted. Diet and lifestyle intervention measures is the mainstay of treatment of NAFLD [102], however, it cannot be successfully or sustainably implemented in most patients. The patients who fail to benefit from lifestyle intervention or those with already advanced disease (significant fibrosis), need pharmacological treatments which are specifically aimed at improving hepatic inflammation, fibrosis and steatohepatitis. Resmetirom, a thyroid hormone receptor β (THR-β) agonist which acts by the activation of the THR receptor in hepatocytes to decrease de novo lipogenesis, enhance fatty acid oxidation, regulate mitophagy and mitochondrial biogenesis, and influence cholesterol metabolism, while also providing direct anti-inflammatory and anti-fibrotic benefits, recently emerged as the first approved drug for the effective management of NAFLD. It is also anticipated to yield better results when combined with other agents like GLP-1RA, albeit under close surveillance [103, 104].

In clinical trials, typical pharmacological interventions for NAFLD focus on regulating glucose and lipid metabolism to safeguard liver health and reduce inflammation. The drugs currently under trials are targeted therapies which can be classified based on their pharmacodynamics as drugs targeting genetics, epigenetics, lipid, carbohydrate, and bile acid metabolism, oxidative stress, inflammation and fibrosis (Table 4). Drugs like semaglutide and sodium-glucose cotransporter-2 inhibitors (SGLT-2i) also demonstrate efficacy in NASH resolution and improvements in liver fat content and liver enzyme levels [105], while Vitamin E supplementation has the tendency to decrease overall mortality and transplant rates, rates of hepatic decompensation, and may benefit both in patients with or without T2DM [106]. Other drugs like Glucagon-like peptide-1 receptor agonists (GLP-1RAs), Biguanides, Thiazolidinediones, Lipid-lowering drugs like statins and ezetimibe, anti-hypertensive drugs like telmisartan, hepatoprotective drugs like ursodeoxycholic acid (UDCA) and silymarin, probiotics, cyclophilin inhibitor, Peroxisome proliferation-activated receptor agonist, other THR-β agonists, incretins, acetyl-CoA carboxylase inhibitors, Diacylglycerol acyltransferase 2 inhibitors (Firsocostat), Stearoyl-CoA desaturase 1 inhibitors**,**Chemokine receptor antagonists (Cenicriviroc), antioxidants, Farnesoid X receptor agonists (Obeticholic acid), Fibroblast growth factor analogs, and others like TVB2640, a novel lipase synthesis inhibitor, have shown promising outcomes but still require further studies [19, 107, 108].

**Table 4:** Overview of various drug classes under investigation for treatment of NAFLD [19, 105–109]

Drug Class	Mechanism of Action	Potential Adverse Effects or Safety Concerns
Glucagon-like peptide-1 receptor agonists (GLP-1RAs)	Increase insulin secretion, decrease glucagon release, slow gastric emptying, and promote satiety	Gastrointestinal symptoms (nausea, vomiting), risk of pancreatitis
Sodium-glucose cotransporter-2 inhibitors (SGLT-2i)	Reduce glucose reabsorption in kidneys, promoting glucose excretion in urine	Genital infections, urinary tract infections, dehydration, possible increased risk of ketoacidosis
Vitamin E	Antioxidant properties reducing oxidative stress in liver	Potential increased risk of haemorrhagic stroke and prostate cancer
Biguanides (e.g., Metformin)	Improve insulin sensitivity, decrease hepatic glucose production	Gastrointestinal upset, risk of lactic acidosis (rare)
Thiazolidinediones (e.g., Pioglitazone)	Improve insulin sensitivity, reduce hepatic fat content	Weight gain, oedema, risk of heart failure, bone fractures
Lipid-lowering drugs (e.g., Statins, Ezetimibe)	Reduce cholesterol synthesis (statins), inhibit cholesterol absorption (ezetimibe)	Muscle pain, liver enzyme abnormalities, risk of diabetes (statins)
Anti-hypertensive drugs (e.g., Telmisartan)	Angiotensin receptor blocker reducing blood pressure and improving insulin sensitivity	Dizziness, hyperkalaemia, renal impairment
Hepatoprotective drugs (e.g., Ursodeoxycholic acid)	Improve bile flow and reduce liver enzyme levels	Diarrhoea, weight gain, potential for liver toxicity in high doses
Probiotics	Modulate gut microbiota, reduce intestinal permeability, and systemic inflammation	Gastrointestinal symptoms (bloating, gas), infection risk in immunocompromised individuals
Cyclophilin inhibitors	Reduce inflammation and fibrosis by inhibiting cyclophilin D	Potential for drug interactions, renal toxicity, gastrointestinal symptoms
Peroxisome proliferation-activated receptor agonists (PPAR agonists)	Improve lipid metabolism, reduce inflammation and fibrosis	Weight gain, fluid retention, risk of cardiovascular events
Thyroid hormone receptor β (THR-β) agonists	Increase fatty acid oxidation, reduce lipogenesis	Potential cardiovascular risks, effects on bone density
Acetyl-CoA carboxylase inhibitors	Inhibit fatty acid synthesis, promote fatty acid oxidation	Gastrointestinal symptoms, liver enzyme abnormalities
Diacylglycerol acyltransferase 2 inhibitors (Firsocostat)	Inhibit triglyceride synthesis	Gastrointestinal symptoms, liver enzyme abnormalities
Stearoyl-CoA desaturase 1 inhibitors	Reduce triglyceride synthesis	Potential for liver enzyme elevations, gastrointestinal symptoms
Chemokine receptor antagonists (Cenicriviroc)	Block chemokine receptors involved in inflammation and fibrosis	Gastrointestinal symptoms, headache, increased risk of infections
Farnesoid X receptor agonists (Obeticholic acid)	Regulate bile acid synthesis, reduce hepatic inflammation and fibrosis	Pruritus, increased cholesterol levels, potential for liver enzyme elevations
Fibroblast growth factor analogs	Regulate metabolism, reduce inflammation and fibrosis	Potential cardiovascular risks, injection site reactions
TVB2640 (lipase synthesis inhibitor)	Inhibit lipase synthesis, reducing liver fat accumulation	Gastrointestinal symptoms, liver enzyme elevations
Natural products (e.g., Terpenoids)	Regulate lipid metabolism, reduce oxidative stress and inflammation	Limited human studies, potential for unknown side effects
Nanotechnology-based treatments	Deliver drugs directly to liver cells	Concerns about long-term safety, immunogenicity, and stability

Natural products such as terpenoids have also being exploited mostly in animal studies for its potentials in treatment of NASH. Though no clinical studies have been initiated, terpenoids play a therapeutic role in NAFLD, mainly by regulating lipid metabolism disorder, insulin resistance, oxidative stress, and inflammation. The AMPK, PPARs, Nrf-2, and SIRT 1 pathways are the main targets for terpenoid treatment. They are promising drugs and will potentially create more opportunities for the treatment of NAFLD [109]. Another promising option is berberine (BBR) which enhances the insulin signaling pathway via several mechanisms, increases glucose disposal by increasing the expression of glucose transporter (GLUT4) on peripheral cells, as well as exertion of metformin-like effects. It exerts some antihyperlipidemic effects and have been shown to poses potent hypotensive and cardioprotective properties while also exerting a lipid lowering effect by modulating gut microbiome and suppressing NAFLD by lowering AST and ALT levels, and improving levels of indirect markers of hepatosteatosis [110, 111].

Nanotechnology is an exciting frontier in medical research. New nanotechnology-based treatments including nano emulsions, liposomes, micelles, polymeric nanoparticles, nanogels, inorganic nanoparticles, and zinc oxide nanoparticles. These nanoparticles could deliver drugs directly into the liver. Despite the optimism surrounding the nanotechnological approach, concerns about long term safety, immunogenicity and stability are still a big concern. Hence, requires further evaluation to overcome these limitations in order to achieve translation into clinical application of these approach [112].

Combination therapies or multi-modal approaches present more avenues for addressing the complex pathogenesis of NAFLD and MetS. By targeting multiple aspects of disease pathology simultaneously, these approaches may offer synergistic benefits and improved outcomes compared to single-target interventions. Recent studies have shown that combining pharmacological treatments like pioglitazone with bariatric surgery is one of the most effective treatment options for patients with NAFLD and MetS [113]. Additionally, studies suggest that combining various drugs targeting insulin resistance with agents modulating lipid metabolism or reducing oxidative stress may demonstrate complementary effects in improving liver function and metabolic parameters although many trials are still ongoing in this regard [114].

## Future directions

Further understanding and management of NAFLD within the background of MetS encompass several key areas of exploration and innovation. Firstly, precision medicine approaches hold significant promise in individualizing interventions based on patient’s unique biochemical characteristics and integrating omics data with clinical parameters to develop personalized treatment strategies but this approach is still in its early stages of development [115]. Secondly, the identification of novel therapeutic targets remains a focal point for pharmacological intervention, with ongoing research aimed at unravelling more molecular mechanisms underlying hepatic lipid accumulation, inflammation, and fibrosis. For instance, newer peroxisome proliferator-activated receptors (PPARs) hold great promise as a target for treatment of NAFLD; PPARs such as pemafibrate, elafibranor, saroglitazar and lanifibranor are currently on the late phase of clinical trials [116]. Additionally, exploring the roles of gut microbiota modulation, bile acid metabolism, and mitochondrial function may unveil new avenues for therapeutic development.

Genetic-based therapies such as micro-RNA, stem-cell based therapy, apoptosis inhibitor-based therapy, and targeted therapeutic strategy of ferroptosis are also under investigation for possible future hope of NAFLD cure [117–119]. Moreover, advancements in non-invasive diagnostic modalities, such as imaging techniques and emerging biomarkers, offer potential for improved accuracy in diagnosing and staging NAFLD without invasive procedures like liver biopsy. Ongoing research into pharmacological interventions targeting glucose and lipid metabolism, along with investigations into novel therapeutic agents spanning various drug classes, presents exciting opportunities for enhancing NAFLD management [114]. Targeting the most appropriate therapies to those most likely to benefit will be critical, especially as many off-label therapies being studied (e.g., GLP-1 agonists) are expensive.

There are some challenges and limitations to the implementation of these approaches. Firstly, large-scale data integration from omics technologies and clinical parameters is essential but poses logistical and computational challenges. Ethical considerations regarding data privacy and consent also need to be addressed to ensure patient confidentiality. Additionally, the availability of appropriate infrastructure and expertise for data analysis and interpretation remains a barrier in many healthcare settings. Furthermore, there is need for improvements to overcome the challenges faced by the use of digital health technologies may be effective in the management of NAFLD and MetS [99, 120, 121]. These technologies require careful evaluation for efficacy, reliability, and accessibility.

## Conclusion

The rising prevalence of obesity worldwide is closely linked to the increasing incidence of Non-Alcoholic Fatty Liver Disease (NAFLD), a hepatic manifestation of metabolic syndrome characterized by complex interactions between visceral adiposity, insulin resistance, and dyslipidaemia. Current diagnostic methods for NAFLD primarily involve imaging techniques like ultrasound and non-invasive elastography, with liver biopsies serving as the gold standard. Emerging biomarkers and OMIC technologies promise enhanced diagnostic accuracy but face challenges in routine clinical implementation due to costs and technical requirements. Treatment options mostly focus on lifestyle interventions, with pharmacological treatments and metabolic surgery showing effectiveness for advanced cases. Notably, Resmetirom has emerged as an effective breakthrough therapy for NAFLD. Future directions emphasize the development of precision medicine, novel therapeutic targets, and improved non-invasive diagnostic tools, aiming to overcome existing barriers and improve patient outcomes.

## Data Availability

Not applicable.

## Abbreviations

AF: Atrial Fibrillation
ALT: Alanine Aminotransferase
apoB100: Apolipoprotein B100
AST: Aspartate Aminotransferase
AUROC: Area Under the Receiver Operating Characteristic Curve
BMI: Body Mass Index
CAD: Coronary Artery Disease
CAP: Controlled Attenuation Parameter
CD36: Cluster of Differentiation 36
ChREBP: Carbohydrate Regulatory Element-Binding Protein
CK-18: Cytokeratin-18 Fragments
CKD: Chronic Kidney Disease
COPD: Chronic Obstructive Pulmonary Disease
cT1: Contrast-Enhanced T1
CV: Cardiovascular
DNL: De Novo Lipogenesis
DTx: Digital Therapy
ELF: Enhanced Liver Fibrosis
FABP: Fatty Acid-Binding Proteins
FAO: Fatty Acid Oxidation
FATP: Fatty Acid Transport Proteins
FFAs: Free Fatty Acids
FIB-4: Fibrosis-4 Index
FNI: Fibrotic NASH Index
GGT: Gamma-Glutamyl Transferase
GERD: Gastrointestinal Reflux Disease
HA: Hyaluronic Acid
HDL: High-Density Lipoprotein
HSI: Hepatic Steatosis Index
HTN: Arterial Hypertension
IL-1β: Interleukin-1 Beta
MAC: Mitral Annular Calcification
M30: Cytokeratin-18 Fragment M30
M65: Cytokeratin-18 Fragment M65
MDD: Major Depressive Disorder
MetS: Metabolic Syndrome
miR34a: MicroRNA 34a
MRI: Magnetic Resonance Imaging
MRE: MR Elastography
MASLD: Metabolic dysfunction-associated steatotic liver disease
MASH: Metabolic dysfunction-associated steatohepatitis
MTTP: Microsomal Triglyceride Transfer Protein
NAFLD: Non-Alcoholic Fatty Liver Disease
NFS: NAFLD Fibrosis Score
NIS-4: NAFLD Inflammatory Score-4
NPV: Negative Predictive Value
OMIC: Genomics, Epigenomics, Transcriptomics, Proteomics, Metabolomics
OSAS: Obstructive Sleep Apnea Syndrome
PCOS: Polycystic Ovary Syndrome
PPARα: Peroxisome Proliferator-Activated Receptor Alpha
PPV: Positive Predictive Value
ROS: Reactive Oxygen Species
sdLDL: Small Dense LDL
SREBP1c: Sterol Regulatory Element-Binding Protein 1c
TG: Triglycerides
TIMP-1: Tissue Inhibitor of Metalloproteinase-1
THR: Thyroid hormone receptor
UDCA: Ursodeoxycholic Acid
USS: Ultrasonography
VA: Ventricular Arrhythmia
VCTE: Vibration Controlled Transient Elastography (FibroScan®)
VLDL: Very Low-Density Lipoproteins
